# The effect of different fuels and clads on neutronic calculations in a boiling water reactor using the Monte Carlo method

**DOI:** 10.1038/s41598-020-79236-8

**Published:** 2020-12-17

**Authors:** Mehtap Düz, Selcan İnal

**Affiliations:** 1 Physics Department, Science and Art Faculty, İnönü University, Malatya, Turkey; 2 Institute of Science, İnönü University, Malatya, Turkey

**Keywords:** Nuclear fuel, Nuclear fusion and fission, Nuclear waste, Theoretical nuclear physics, Physics

## Abstract

In this study, a Boiling Water Reactor (BWR) modeling was done for the reactor core divided into square lattice 8 × 8 type using the Monte Carlo Method. Each of the square lattices in the reactor core was divided into small square lattices 7 × 7 type in groups of four. In the BWR designed in this study, modeling was made on fuel assemblies at pin-by-pin level by using neptunium mixed fuels as fuel rod, Zr-2 and SiC as fuel cladding, H_2_O as coolant. In fuel rods were used NpO_2_ and NpF_4_ fuels at the rate of 0.2%-1% as neptunium mixed fuels. In this study, the effect on the neutronic calculations as k_eff_, neutron flux, fission energy, heating of NpO_2_ and NpF_4_ fuels in 0.2%-1% rates, and Zr-2 and SiC clads were investigated in the designed BWR system. The three-dimensional (3-D) modelling of the reactor core and fuel assembly into the designed BWR system was performed by using MCNPX-2.7.0 Monte Carlo method and the ENDF/B-VII.0 nuclear data library.

## Introduction

Light water reactors (LWR) developed in the 1950s; it is the most common fission reactor that uses fissile material as fuel and normal water as both coolant and neutron moderator^1,2^. The boiling water reactor (BWR) used mainly for the production of electrical energy based on pressure is a kind of light water nuclear reactor. All of the nuclear reactors available today are fission reactors, and the spent fuel from these reactors includes uranium (about 95 wt%), plutonium (0.9 wt%), minor actinides; Np, Am and Cm (0.1 wt%) and fission products such as Cs, Sr, Tc and I (4 wt%)^3,4^. These wastes, which may have a high radiotoxicity and a good source of energy remaining from the existing reactors, are stored for future use. However, these wastes should be transformed into stable and short-lived isotopes by nuclear reactions such as fission or neutron capture. Thus, solutions will be produced for both environmental and fuel problems that will occur in the near future^5–8^.

Zircaloy-2 (Zr-2) and SiC–SiC ceramic matrix composites (CMCs) were developed as a fuel cladding in light water reactors (both BWR and PWR). Cracking in the fuel cladding occurs by a combination of cladding stresses and the corrosive effects of some fission products such as iodine and cadmium^9^. The cracking of fuel cladding for reactor life and power generation is undesirable. Zr-2 and SiC have the characteristics that cracks do not spread through the cladding during a power ramp, their irradiation stability, the stress level low^10–12^, the ability to maintain their mechanical properties and chemical inactivity at high temperatures^13^, and exceptional resistance to steam oxidation^9,11,12^. In order to increase nuclear energy production from nuclear fuel, it is desired to have low thermal neutron absorption cross section values ​​such as 0.18 barn and 0.12 barn for Zr-2 and SiC in the fuel cladding material selection, respectively^14^. Therefore, Zr-2 (98% Zr, 1.6% Sn, 0.15% Fe, 0.1% Cr, 0.05% Ni)^15,16^ and SiC (48.3% Si, 51.7% C)^17^ were used as fuel cladding in this study. Minor actinides are valuable but can be dangerous if used incorrectly. Therefore, neptunium-added radioactive materials were used in this study to reduce the amount of minor actinides. It was designed a BWR system using NpO_2_ and NpF_4_ fuels at the rate of 0.2–1% as neptunium mixed fuels, and Zr-2 and SiC as clad in the current study. The purpose of this study was to investigate the effect of the neptunium mixed fuels and clads on three-dimensional (3-D) neutronic calculations, such as k_eff_, neutron flux, fission energy and heating in the designed BWR system. The 3-D modelling of the reactor core and fuel assembly into the designed BWR system was performed by using MCNPX-2.7.0 Monte Carlo method and the ENDF/B-VII.0 nuclear data library.

## Method

### Core geometry and fuel assembly geometry

**Figure 1:**
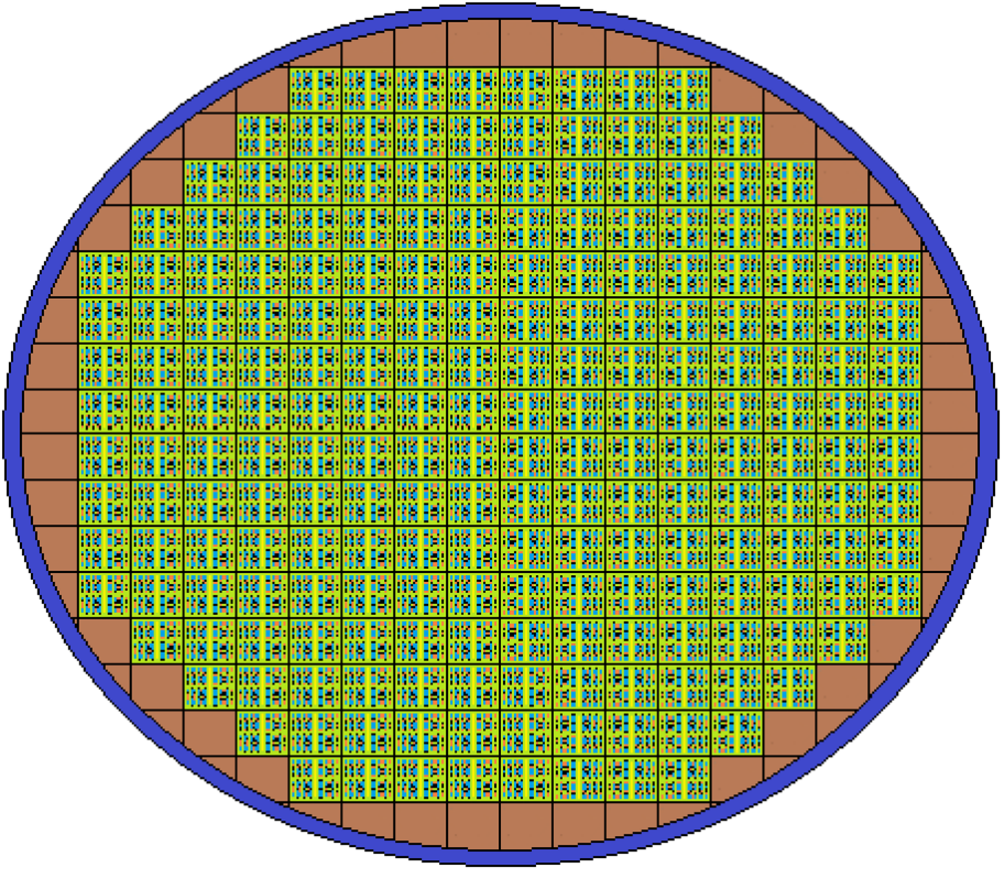
The core design of the designed BWR system. (MCNPX Vised, version 2.7.0, https://mcnp.lanl.gov).

In this study, Peach Bottom-2 nuclear power plant^18^ was used for the selection of design parameters values of BWR in our model. BWR design parameters values of this study are shown in Table 1. The core design of the cylindrical BWR that we modeled in MCNPX is shown in Fig. 1. Moreover, as shown in Fig. 1, the reactor core is divided into the square lattice 8 × 8 type. The constant pitch of the square lattice 8 × 8 type is 30.48 cm. The core was surrounded by a graphite reflector. The outboard side of the reflector was surrounded by SS316LN ferritic steel.

**Table 1:** The core information of the designed BWR system.

Reactor power (MW)	2000
Radius of the cylinder (cm)	264.08
Core height (cm)	365.76
Ferritic steel width (cm)	5
Fuel assemblies number	185
Small square region size (cm)	13.40612
Fuel rod radius (cm)	0.60579
Clad radius (cm)	0.71501
Gap width (cm)	0.01524
Total fuel rod number	36,260
Total cruciform number	185
Absorber pins radius (cm)	0.23876

**Figure 2:**
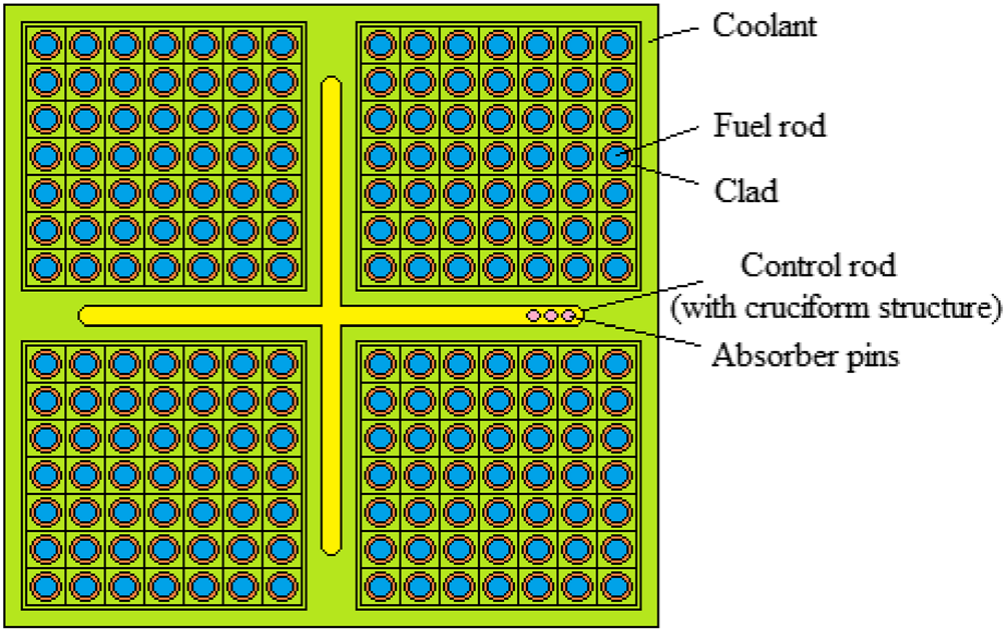
The square lattice in the core of the designed BWR system. (MCNPX Vised, version 2.7.0, https://mcnp.lanl.gov).

The core surrounded with approximately 40 reflector assemblies. As shown in Fig. 2, the fuel rods were put into square lattices and every square lattice was divided into four small square regions. Every small square region was divided into 7 × 7 type the small square lattices. The constant pitch of the small square lattice 7 × 7 type is 1.94084 cm.

**Figure 3:**
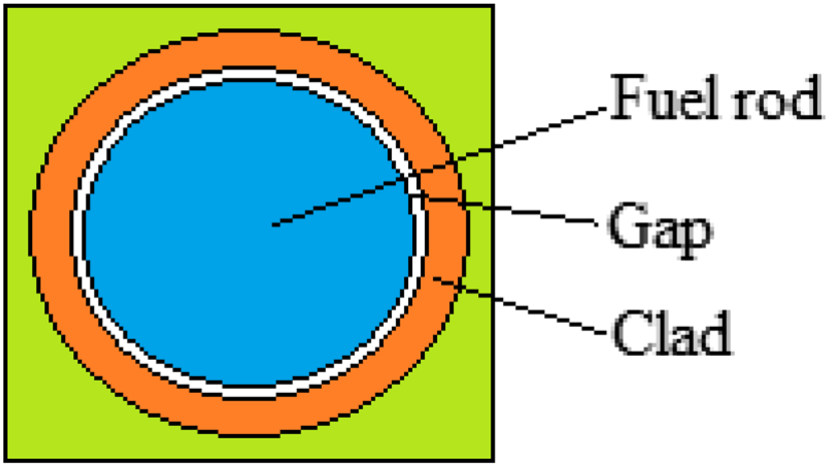
Pin cell geometry. (MCNPX Vised, version 2.7.0, https://mcnp.lanl.gov).

Cylindrical fuel pins were placed in the small square lattices. The fuel pins were created from the fuel rod, gap and clad. The pin cell geometry in the small square lattice of the designed BWR system is shown in Fig. 3^19^. 49 fuel rods inside every small square lattice and 196 fuel rods inside every square lattice were placed in the designed BWR system. 0.2–1% NpO_2_ and NpF_4_ were filled into the fuel rods and Zircaloy-2 and SiC were used as clad in this study.

As seen in Fig. 2, the control rods used to ensure reactivity control were placed in cruciform between four small square lattices. The blade radius of the control rod is 0.39624 cm and the blade half length is 11.98626 cm. The control rods were filled by B_4_C in the designed BWR system. The absorber pins were made in cylinder shape into the cruciform. In the every cruciform were used total 84 absorber pins (21 per wing). Thus, it was used total 15,540 absorber pins in the designed BWR system. Type-304 stainless steel was used as structural material in the cruciform. H_2_O was used as coolant in the designed BWR system.

### The system modeling code

Nuclear data are important parameters for reactor physics modeling and simulation. Nuclear data can be obtained by experimental measurement, theoretical calculation and Evaluated Nuclear Data Files (ENDFs). ENDFs have been separately released from different countries to standardize as internationally experimental data and calculations^20–24^. Internationally accepted ENDFs are used with model calculations.

In this study, ENDF/B-VII.0^25^ published in 2006 was used for ENDF/B from evaluated nuclear data files for Monte Carlo N-Particle (MCNP) method^26,27^.

The Monte Carlo method is generally used because of the complex three-dimensional configuration of the materials, reactor physics modeling and simulation, and the many physics problems of deterministic methods. MCNPX (MCNP eXtended)^28^, which the combination of MCNP and LAHET^29^ codes is a Monte Carlo radiation transport code that tracks all particles at almost any energies. The MCNPX transport code uses the continuous energy cross-sections^30^ to transport low-energy particles (< 20 meV), while it uses cross section libraries for low energy particles (< 150 meV) and nuclear models for high energy particles (> 150 meV)^31^. The MCNPX uses standard cross-section libraries compiled from ENDF/B for neutron, proton and photonuclear interactions. Different intranuclear, preequilibrium and evaporation-fission models have been implemented into MCNPX-2.7.0 version, which offers seven different options based on four physics packages: Bertini^32,33^ and ISABEL^34,35^, INCL4^36–38^, the CEM2k^39^ package and two evaporation-fission models Dresner^40^, ABLA^41^. Bertini, ISABEL, and INCL4 are INC models, which can be coupled with ABLA and Dresner evaporation–fission codes. CEM2k is a cascade-preequilibrium-evaporation model^42,43^. The three-dimensional (3-D) modelling of the reactor core and fuel assembly into the designed BWR system was performed by using MCNPX-2.7.0 Monte Carlo method and the ENDF/B-VII.0 nuclear data library.

## Results and discussion

### Effective neutron multiplication factor

The effective neutron multiplication factor (k_eff_) plays an extremely important role in determining nuclear reactor behavior. The criticality factor k_eff_ is effective in determining the contribution of nuclear reactions to neutron multiplication. k_eff_ is defined as the net increase in the number of neutrons from one generation to the next (Eq. 1). k_eff_ = 1 is the desired critical operating mode of a reactor. If k_eff_ < 1, the number of neutrons will decrease exponentially. If k_eff_ > 1, the number of neutrons will increase exponentially, which will be dangerous to operate the reactor^44,45^.

1 $${{\mathrm{k}}_{\mathrm{eff}}=\frac{(\mathrm{number of neutrons generated in the next generation})}{(\mathrm{number of neutrons generated in a generation})}}$$

**Figure 4:**
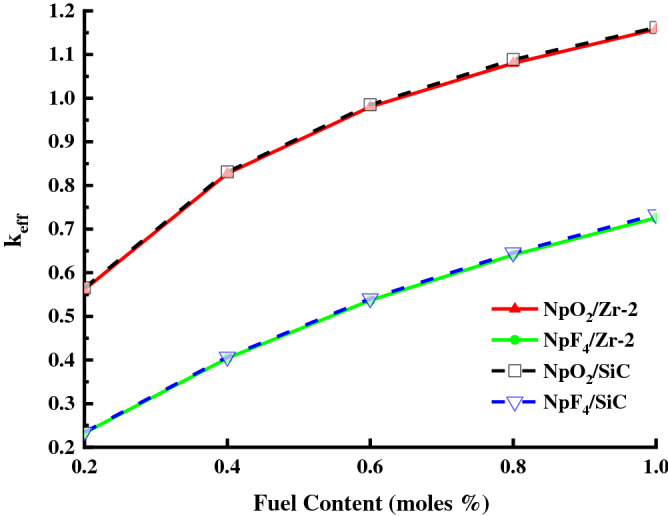
The k_eff_ values for Zr-2 and SiC clads, the fuel components NpO_2_ and NpF_4_ in the BWR system. (Origin 2018, version 9.5, www.originlab.com).

In this study, k_eff_ was examined for Zr-2 and SiC as clad and NpO_2_ and NpF_4_ fuels as Neptunium Mixed Fuels. Figure 4 shows the k_eff_ value for the Zr-2 and SiC clad at 0.2–1% relative to the NpO_2_ and NpF_4_ fuel compositions. The effective multiplication constant must k_eff_ ≤ 1 in the designed BWR system to avoid the critical accident. As shown in Fig. 4, the k_eff_ value increases as the NpO_2_ and NpF_4_ fuel contents ratios increase from 0.2% to 1%. Figure 4 shows that the lower and upper k_eff_ limit values of 0.6–0.8% NpO_2_ are 0.98033–1.08004 for Zr-2, and those of 0.6–0.8% NpO_2_ are 0.98517–1.08856 for SiC clads, respectively. Table 2 shows the calculated k_eff_ values ​​for three different fuel ratios of NpO_2_ and NpF_4_ between 0.6–0.8% in Zr-2 and SiC clads. As shown in Fig. 4 and Table 2, the k_eff_ values for Zr-2 and SiC clads of NpO_2_ fuel, and k_eff_ values for Zr-2 and SiC clads of NpF_4_ fuel are similar because of the similar thermal neutron absorption cross sections of Zr-2 (σ = 0.18 b) and SiC (σ = 0.12 b) clads values. Moreover, for the fuel ratios used, the k_eff_ values obtained from SiC are higher than those of Zr-2. As a conclusion, the calculated k_eff_ value for 0.6–0.8% NpO_2_ fuel and SiC clad provided the desired (k_eff_ ≤ 1) critical value. Therefore, considering the fuel ratios (0.6–0.8%) for which the k_eff_ critical value was obtained, the lower limit of the fuel ratio was determined as 0.2% for below 0.6%, and the upper limit as 1% for above 0.8%.

**Table 2:** k_eff_ values for Zr-2 and SiC clads, the fuel components NpO_2_ and NpF_4_ in the BWR system.

Fuel content (moles %)	Zr-2		SiC	
	NpO_2_	NpF_4_	NpO_2_	NpF_4_
0.65	1.00822	0.56500	1.01502	0.56508
0.7	1.03770	0.59059	1.04158	0.59649
0.75	1.06133	0.61686	1.06745	0.62200

## Neutron flux

The neutron flux distribution in a nuclear reactor core is important for neutronic calculations of all neutron-induced nuclear reactions such as fission energy, heating, fissile fuel production. Neutron flux is the total length travelled by all neutrons per unit time and volume^46^. The process of neutron transport should be investigated to determine the neutron flux distribution in the reactor. For this purpose, Boltzmann equation also called the neutron transport equation^46,47^ is commonly used to calculate neutron flux in a reactor.

2 $$\frac{1}{v}\frac{\partial \varphi}{\partial t}+ \Omega .\nabla\varphi +\sum_{t}\left(r, E\right)\varphi = q(r, E, \Omega ,t)$$

3 $$\varphi = \varphi (r,E,\Omega ,t)$$

$\frac{1}{v}\frac{\partial \varphi}{\partial t}$ = Change of neutron flux in unit time, $\Omega .\nabla\varphi$ = Neutron loss because of convection, $\sum_{t}\left(r, E\right)\varphi$= Neutron loss because of nuclear reactions.

Terms in Eq. (2) for $q(r, E,\Omega , t)\varphi$ can be defined as follows (Eq. 4):

4 $${\text{q}}\left( {{\text{r}},{\text{E}},\Omega ,{\text{t}}} \right) = \smallint _{{4\pi }} {\text{d}}\Omega ^{\prime } \int_{0}^{\infty } {{\text{dE}}^{\prime } } \sum _{s} {\text{(E}}^{\prime } \to E,\Omega ^{\prime } \to \Omega )\varphi ({\text{r}},E^{\prime } ,\Omega ^{\prime } ,t) + s({\text{r}},E,\Omega ,{\text{t}})$$

$\int_{{4\pi }} {d\Omega ^{\prime } } \int_{0}^{\infty } {dE^{\prime } } \sum _{s} (E^{\prime } \to E,\Omega ^{\prime } \to \Omega )\varphi (r,E^{\prime } ,\Omega ^{\prime } ,t)$ = Contribution of neutrons on neutron flux due to scattering, = $s(r, E,\Omega , t)$Contribution of neutron source independent on the neutron flux.

In this study, neutron flux distribution was calculated using MCNPX-2.7.0 code and ENDF/B-VII.0 to solve Boltzmann Eqs. (2)^46,47^ and (4)^46,47^. F4 tally was used to calculate the neutron flux distribution by track-length estimates of the total cell flux. Since neutron flux distribution is an important parameter in evaluating the neutronic performance of a reactor, neutron flux distribution for different clad and fuels was calculated in this study.

**Figure 5:**
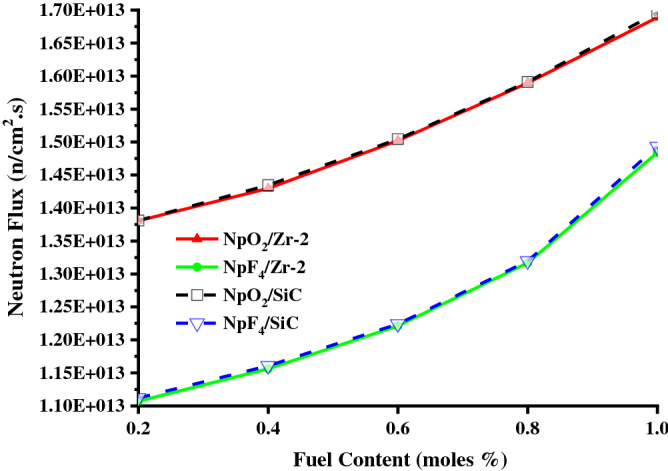
The neutron flux values for Zr-2 and SiC clads, the fuel components NpO_2_ and NpF_4_ in the BWR system. (Origin 2018, version 9.5, www.originlab.com).

Figure 5 shows that the neutron flux value for Zr-2 and SiC clads increases as the NpO_2_ and NpF_4_ fuel content ratios increase from 0.2% to 1%. As seen in Fig. 5 (for SiC captures less thermal neutrons than Zr-2), the highest neutron flux (1.696.10^13^n/cm^2^.s) result from 1% NpO_2_ fuel for SiC clad and the lowest neutron flux (1.107.10^13^n/cm^2^.s) result from 0.2% NpF_4_ fuel for Zr-2 clad.

### Fission energy

Almost all fast neutrons in a nuclear reactor are obtained by fission reactions. Fission energy is produced by fission reactions. The fission energy released consists of various energy modes, such as kinetic energy from fission products and fission neutrons, fast gamma rays and energy from subsequent neutron capture^43,48^. Fission energy was calculated using F7 tally. Fission energy is an important parameter for neutronic calculations of a nuclear reactor.

**Figure 6:**
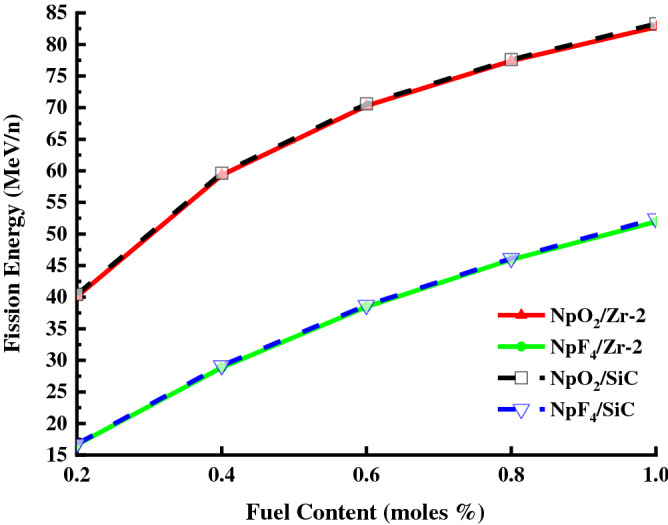
The fission energy values for Zr-2 and SiC clads, the fuel components NpO_2_ and NpF_4_ in the BWR system. (Origin 2018, version 9.5, www.originlab.com).

Figure 6 shows the calculated fission energy values for Zr-2 and SiC clads, and NpO_2_ and NpF_4_ fuel content ratios (0.2–1%) in the designed BWR system. The fission energy values increased as the NpO_2_ and NpF_4_ fuel content ratios increase from 0.2% to 1%, for both Zr-2 and SiC clads. Since the thermal neutron cross section of Zr-2 is larger than SiC, fewer thermal neutrons in the Zr-2 cladding will contribute to fission energy generation. Hence, as seen in Fig. 6, the highest fission energy value (83.28 meV/n) was obtained from 1% NpO_2_ fuel for SiC clad and the lowest fission energy value (16.64 meV/n) was obtained from 0.2% NpF_4_ fuel for Zr-2 clad.

### Heating

Neutron flux distribution and neutron multiplicity per incident neutron determine the performance of the nuclear reactor system. Therefore, the contribution of neutron spectrum and neutron multiplicity to heat energy production should be determined in the nuclear system. Moreover, heating expressed as heat energy production is produced through neutron flux, fission and other reactions. Most of the fission energy of the nuclear reactor, especially in the fuel zone, is converted into heating. A small heat release will occur through neutron and γ-ray radiation in the coolant around the fuel rods^49,50^. F6 tally was used to calculate the heating by track-length heating of the total cell heating.

**Figure 7:**
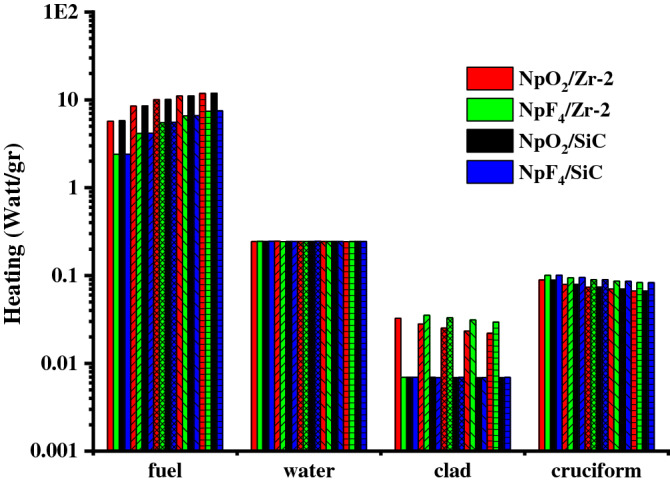
The contribution of each zone to the heating for Zr-2 and SiC clads, the fuel components NpO_2_ and NpF_4_ ( 0.2%,  0.4%,  0.6%,  0.8%,  1%) in the BWR system. (Origin 2018, version 9.5, www.originlab.com).

Figure 7 shows the heating values calculated in the relevant regions of the designed BWR system for both Zr-2 and SiC clads, and NpO_2_ and NpF_4_ fuel contents (0.2–1% rates). In this study, neutron flux in fuel region is more intense than other regions, since fission reaction occurs in Np additive fuel rods in the fuel region of the designed reactor. For this reason, as seen in Fig. 7, the heating value increases as the NpO_2_ and NpF_4_ fuel content increase from 0.2% to 1% in the fuel region where the neutron flux is intense (for Zr-2, SiC clads). When Fig. 7 is examined for the fuel region, it is seen that the highest contribution to heating comes from 1% NpO_2_ with values ​​of 11.85911 W/gr for Zr-2 and 11.93478 W/gr for SiC, while the lowest contribution to heating comes from 0.2% NpF_4_ with values ​​of 2.40284 W/gr for Zr-2 and 2.40285 W/gr for SiC. As a result, the heating value in the fuel region for 1% NpO_2_ fuel content and SiC clad is higher than other fuel content ratios and clads. The heating values of the water region (coolant) shown in Fig. 7 are presented in detail in Table 3. The heating value generated in the water region around the fuel rods through neutron and γ-ray radiation with fission products is smaller than in the fuel region. As shown in Table 3, the heating value in the water region increased slightly with the increase of NpO_2_ and NpF_4_ fuel content ratios from 0.2% to 1% for Zr-2 and SiC clads. Moreover, as the highest contribution to heating in the water region comes from 1% NpO_2_ and SiC clad, the lowest contribution comes from 0.2% NpF_4_ and Zr-2 clad. Figure 7 shows that the heating values in the clad and cruciform region decreases as the NpO_2_ and NpF_4_ fuel content ratios increase from 0.2% to 1%, for Zr-2 and SiC clads. For the clad and fuel content ratios, the contributions of the regions to heating from higher to lower value are fuel, water, cruciform and clad, respectively.

**Table 3:** The heating (Watt/gr) of the water region for Zr-2 and SiC clads, the fuel components NpO_2_ and NpF_4_ in the BWR system.

Fuel content (moles %)	Zr-2		SiC	
	NpO_2_	NpF_4_	NpO_2_	NpF_4_
0.2	0.244144	0.243331	0.244304	0.243443
0.4	0.244395	0.243687	0.244463	0.243690
0.6	0.244777	0.243802	0.244991	0.243817
0.8	0.245134	0.243848	0.245135	0.243851
1	0.245136	0.243882	0.246018	0.243975

Table 4 shows the integrated heating for NpO_2_ and NpF_4_ fuel content ratios (0.2–1%), and Zr-2 and SiC clads, in our BWR system. It is seen that the integrated heating value increased due to the increase in the fission reaction resulting from the increase of NpO_2_ and NpF_4_ fuel content from 0.2% to 1%, for Zr-2 and SiC clads. Integrated heating values for Zr-2 and SiC clads of NpO_2_ fuel, and integrated heating values for Zr-2 and SiC clads of NpF_4_ fuel are similar because of the similar thermal neutron absorption cross sections of Zr-2 and SiC clads values. Moreover, when Zr-2 and SiC clads are compared with NpO_2_ and NpF_4_ fuel content, it is seen that the integrated heating value found when using SiC is greater than those of Zr-2. As the highest integrated heating value was obtained from 1% NpO_2_ fuel for SiC clad with 24.51 W/gr, the lowest integrated heating value was obtained from 0.2% NpF_4_ fuel for Zr clad with 5.51 W/gr.

**Table 4:** The integrated heating (Watt/gr) for Zr-2 and SiC clads, the fuel components NpO_2_ and NpF_4_ in the BWR system.

Fuel content (moles %)	Zr-2		SiC	
	NpO_2_	NpF_4_	NpO_2_	NpF_4_
0.2	12.22	5.51	12.27	5.52
0.4	17.71	9.02	17.75	9.05
0.6	20.83	11.76	20.89	11.78
0.8	22.86	13.89	22.89	13.90
1	24.38	15.60	24.51	15.70

## Conclusions

In this study, a BWR system with 8 × 8 type square lattice is designed. Each square lattice was divided into small square lattices of 7 × 7 type, which consist of Zr-2 and SiC clads, 0.2–1% NpO_2_, NpF_4_ fuel rods, water and cruciform. In the study; k_eff_, neutron flux, fission energy, heating were calculated for 0.2–1% NpO_2_, NpF_4_ fuels and Zr-2, SiC clads. In the designed BWR system, these neutronic calculations were made using the MCNPX-2.7.0 Monte Carlo method and ENDF/B-VII.0 nuclear data library.

In the study, it was observed that k_eff_, neutron flux, fission energy, heating values ​​increased with the increasing rates of NpO_2_ and NpF_4_ fuels in both Zr-2 and SiC clads.

It was found that neutronic results calculated with NpO_2_ fuel and SiC clad were higher than NpF_4_ fuel and Zr-2 clad. As a conclusion, considering the neutronic results obtained from k_eff_, neutron flux, fission energy and heating values, it is recommended to use NpO_2_ fuel and SiC clad in BWR reactor models.

## Data Availability

The datasets generated during and/or analyzed during the current study are available from the corresponding author on reasonable request.
